# Interpretable Detection of Diabetic Retinopathy, Retinal Vein Occlusion, Age-Related Macular Degeneration, and Other Fundus Conditions

**DOI:** 10.3390/diagnostics14020121

**Published:** 2024-01-05

**Authors:** Wenlong Li, Linbo Bian, Baikai Ma, Tong Sun, Yiyun Liu, Zhengze Sun, Lin Zhao, Kang Feng, Fan Yang, Xiaona Wang, Szyyann Chan, Hongliang Dou, Hong Qi

**Affiliations:** 1 Department of Ophthalmology, Peking University Third Hospital, Beijing 100191, China; docwenlong@163.com (W.L.); linbobian@163.com (L.B.); doctormbk@bjmu.edu.cn (B.M.); suntongpku@126.com (T.S.); jessssie1012@outlook.com (Y.L.); 1910122424@pku.edu.cn (Z.S.); doczlin@163.com (L.Z.); ocufeng@hsc.pku.edu.cn (K.F.); yangfan@bjmu.edu.cn (F.Y.); 15801635367@163.com (X.W.); szyyann@163.com (S.C.); 2 Beijing Key Laboratory of Restoration of Damaged Ocular Nerve, Beijing 100191, China

**Keywords:** interpretable, automated detection, diabetic retinopathy, retinal vein occlusion, age-related macular degeneration

## Abstract

Diabetic retinopathy (DR), retinal vein occlusion (RVO), and age-related macular degeneration (AMD) pose significant global health challenges, often resulting in vision impairment and blindness. Automatic detection of these conditions is crucial, particularly in underserved rural areas with limited access to ophthalmic services. Despite remarkable advancements in artificial intelligence, especially convolutional neural networks (CNNs), their complexity can make interpretation difficult. In this study, we curated a dataset consisting of 15,089 color fundus photographs (CFPs) obtained from 8110 patients who underwent fundus fluorescein angiography (FFA) examination. The primary objective was to construct integrated models that merge CNNs with an attention mechanism. These models were designed for a hierarchical multilabel classification task, focusing on the detection of DR, RVO, AMD, and other fundus conditions. Furthermore, our approach extended to the detailed classification of DR, RVO, and AMD according to their respective subclasses. We employed a methodology that entails the translation of diagnostic information obtained from FFA results into CFPs. Our investigation focused on evaluating the models’ ability to achieve precise diagnoses solely based on CFPs. Remarkably, our models showcased improvements across diverse fundus conditions, with the ConvNeXt-base + attention model standing out for its exceptional performance. The ConvNeXt-base + attention model achieved remarkable metrics, including an area under the receiver operating characteristic curve (AUC) of 0.943, a referable F1 score of 0.870, and a Cohen’s kappa of 0.778 for DR detection. For RVO, it attained an AUC of 0.960, a referable F1 score of 0.854, and a Cohen’s kappa of 0.819. Furthermore, in AMD detection, the model achieved an AUC of 0.959, an F1 score of 0.727, and a Cohen’s kappa of 0.686. Impressively, the model demonstrated proficiency in subclassifying RVO and AMD, showcasing commendable sensitivity and specificity. Moreover, our models enhanced interpretability by visualizing attention weights on fundus images, aiding in the identification of disease findings. These outcomes underscore the substantial impact of our models in advancing the detection of DR, RVO, and AMD, offering the potential for improved patient outcomes and positively influencing the healthcare landscape.

## 1. Introduction

Diabetic retinopathy (DR) [1], retinal vein occlusion (RVO) [2,3], and age-related macular degeneration (AMD) [4] are the leading causes of vision impairment in various populations [1,4,5], with DR being particularly noteworthy. Of an estimated 285 million people with diabetes mellitus worldwide, approximately one-third have signs of DR, and of these, a further one-third of patients with DR have vision-threatening DR, including diabetic macular edema [6]. Mild nonproliferative diabetic retinopathy (NPDR) represents the initial phase of diabetic retinopathy, marked by the development of microaneurysms. As the condition progresses, proliferative diabetic retinopathy (PDR) emerges as an advanced stage, where individuals may even experience vitreous hemorrhage (VH), posing a significant risk of severe vision impairment [1]. Moreover, RVO is the second most common retinal vascular disorder, affecting 16.4 million people worldwide in 2008. The classification of RVO can be broken down into branch retinal vein occlusion (BRVO), hemiretinal vein occlusion (HRVO), and central retinal vein occlusion (CRVO) depending on the site of the obstruction. However, there is rather less research attention on RVO than on DR, for which abundant studies have been conducted [2,3,7,8]. AMD is highly prevalent among the elderly population and has two main types, nonneovascular (dry AMD) and neovascular AMD (wet AMD) [4]. Individuals diagnosed with these conditions may also experience the onset of macular edema (ME), leading to visual impairment. The progression of DR, RVO, and AMD is often nearly irreversible [9,10,11]; therefore, the timely and accurate diagnosis of these fundus diseases is crucial for facilitating appropriate treatment and preserving or improving patients’ vision. Color fundus photographs (CFPs) are widely utilized for the preliminary screening and diagnosis of fundus diseases, owing to the noninvasive characteristics of color fundus photography and its proficiency in capturing intricate retinal images compared to fundus fluorescein angiography (FFA), which is invasive, challenging to perform, and may induce allergic reactions. The conventional approach to interpretation of CFPs relies on the expertise of professionals who manually analyze the images. However, the escalating incidence of diabetes [12], hypertension [13], and associated ocular conditions has placed a considerable burden on healthcare systems. This is particularly challenging in resource-limited settings where access to specialized ophthalmic services is constrained [14].

In recent years, artificial intelligence (AI) has emerged as a promising tool in the field of medical imaging, offering the potential to automate the analysis of fundus images and assist healthcare professionals in diagnosing retinal diseases accurately and efficiently [15,16,17]. Deep learning techniques, especially convolutional neural networks (CNNs), have exhibited remarkable success in various image recognition tasks, including the analysis of fundus images for DR [15,16,18,19,20,21,22,23,24,25], RVO [26,27,28,29], and AMD [30,31,32,33,34,35,36]. Despite the promising outcomes generated by these techniques, their efficacy is primarily confined to a singular task. Additionally, the intricate nature of CNNs frequently poses challenges in deciphering and understanding the rationale behind their decisions. 

In this study, we address these challenges by proposing integrated models that offer interpretability in automated detection of DR, RVO, AMD, and other fundus conditions. Our study involves translating diagnostic information derived from FFA results to CFPs. We explore the models’ capacity to make accurate diagnoses using only CFPs. Moreover, our approach extends beyond mere detection to encompass the classification of DR, RVO, and AMD into their respective subclasses. We leverage the power of CNNs, enhancing them with an attention mechanism [37]. By incorporating this attention mechanism, our models can highlight specific regions in CFPs, providing valuable insights into the decision-making process.

## 2. Materials and Methods

### 2.1. Data Collection

This study adhered to the tenets of the Declaration of Helsinki, and the protocol was approved by the Ethics Committee of Peking University Third Hospital (approval code: M2023513, approval date: 12 September 2023). Our dataset was compiled at the ophthalmology center of Peking University Third Hospital, covering the period from 12 December 2013 to 29 November 2022. A comprehensive dataset comprising 22,383 CFPs (Digital Retinal Camera, CR-2 AF, Canon, Tokyo, Japan) was gathered through FFA (FF 450 plus, Carl Zeiss Meditec AG, Jena, Germany) examinations performed on 17,647 eyes belonging to 8833 patients. These examinations were conducted by two highly skilled examiners. The resulting images were then interpreted in clinical practice by a panel of 18 proficient ophthalmologists, each holding the esteemed position of attending doctor or higher and possessing profound expertise in fundus analysis. During the collection process, images were anonymized to protect patients’ privacy, and examinations lacking color images or report documents as well as any damaged or broken images were excluded. The last examination of a patient (both eyes included) was adopted, and the image quality of CFPs was assessed using AutoMorph [38], excluding images of poor quality or those containing artifacts. This resulted in a final dataset of 15,089 CFPs (15,089 eyes) from 8110 patients.

Diagnoses were established through the analysis of FFA images, covering a wide spectrum of ocular conditions, including DR, RVO, and AMD. Moreover, within the dataset, prevalent occurrences included ME, VH, and laser spots. In this study, DR, RVO, AMD, ME, VH, and laser spots were considered as primary classes, and a case could simultaneously exhibit these conditions. DR subclasses included NPDR (excluding severe NPDR), severe NPDR (sNPDR), and PDR. RVO subclasses were limited to BRVO and CRVO, with HRVO excluded due to its scarcity in this dataset (91 eyes), while AMD was classified into dry and wet subtypes. It is important to note that less common diagnoses such as hypertensive retinopathy and pathologic myopia were infrequent in the dataset and were not within the scope of this research. Notably, the dataset, sourced from clinical practice, may lack precise subclass information for some entries related to DR, RVO, and AMD.

### 2.2. Model Construction and Configurations

Our tasks involved hierarchical multilabel classification, and we employed advanced CNN architectures, namely ResNet101 [39], EfficientNetV2-M [40], and ConvNeXt-base [41], as the foundation for our models. To enhance their interpretability, we integrated an attention mechanism [37], allowing our models to pinpoint relevant regions in CFPs. 

**Figure 1:**
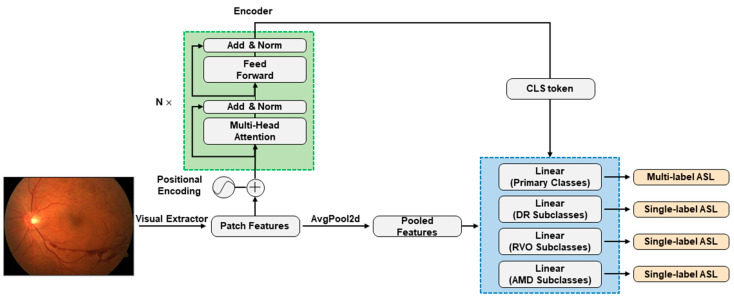
CNN + attention modelling framework. The green dashed box highlights the core of the CNN + attention models, namely, the standard Transformer encoder. Patch features are extracted by the ResNet101, EfficientNetV2-M, or ConvNeXt-base CNN architectures. In pure CNN models, patch features undergo average pooling (AvgPool2d) before reaching multitask classification heads within the blue dashed box. In CNN + attention models, the standard Transformer encoder encodes patch features and forwards CLS token to the final classification heads. The classification heads handle primary classes, observing all training data with multilabel asymmetric loss (ASL) calculation, while the three other heads for subclasses only see data with specified subclass information, calculating individual single-label ASLs. The total loss during training is the sum of multilabel ASL and three single-label ASLs.

Given an image, the pretrained CNNs, are utilized to extract visual features in patches. For CNNs as basic comparisons, these patch features are pooled with AvgPool2d and fed into multitask classification heads, one for predicting the presence of primary classses, and the other three for predicting the subclasses of DR, RVO, and AMD (Figure 1). For our CNN + attention-fused models, these patch features serve as the input for the standard Transformer encoder [37]. The encoder consists of stacked self-attention layers based on multihead attention (MHA). The MHA mechanism is composed of n parallel heads, each defined as scaled dot-product attention:

$$Att_{i}\left(X,Y\right)=softmax\left(\frac{XW_{i}^{Q}{\left(YW_{i}^{K}\right)}^{T}}{\sqrt{d_{n}}}\right)YW_{i}^{V}$$

(1) $$MHA\left(X,Y\right)=\left[Att_{1}\left(X,Y\right);\ldots ;Att_{n}\left(X,Y\right)\right]W^{O}$$

where $X\in {\mathbb{R}}^{\left(l_{x}\times d\right)}$ and $Y\in {\mathbb{R}}^{\left(l_{y}\times d\right)}$ denote the Query matrix and the Key/Value matrix, respectively, and $W_{i}^{Q},W_{i}^{K},W_{i}^{V}\in {\mathbb{R}}^{\left(d\times d_{n}\right)}$ and $W^{O}\in {\mathbb{R}}^{\left(d\times d\right)}$ are learnable parameters, where $d_{n}=d/n$ and $\left[.;.\right]$ stands for the concatenation operation. We extracted the classification (CLS) token, a condensed representation of the encoded visual features. This token was then forwarded through the identical multitask classification heads to predict both primary classes and their respective subclasses if applicable (Figure 1).

To address data imbalance for multilabel classification effectively, we employed an asymmetric loss (ASL) function [42]. Given a set of K labels, the network produces an individual logit designated as $z_{k}$ for each label. These logits subsequently undergo activation through separate sigmoid functions, denoted as $\sigma \left(z_{k}\right)$ . Assuming $y_{k}$ represents the true value for class k , the comprehensive classification loss $L_{tot}$ is formulated by summing the binary losses across all K labels:

(2) $$L_{tot}=\overset{K}{\underset{k=1}{\sum }}L\left(\sigma \left(z_{k}\right),y_{k}\right)$$

The binary loss per label, denoted as L , can be expressed in a general form as follows:

(3) $$L=-yL_{+}-\left(1-y\right)L_{-}$$

The components $L_{+}$ and $L_{-}$ correspond to the positive and negative parts, respectively. The ASL function is defined as follows:

(4) $$ASL=\left\{\begin{array}{c} L_{+}={\left(1-p\right)}^{\gamma_{+}}log\left(p\right)\;\;\;\;\;\;\\ L_{-}={\left(p_{m}\right)}^{\gamma_{-}}log\left(1-p_{m}\right) \end{array}\right.$$

where $p=\sigma \left(z\right)$ , with the class index k omitted, represents the network’s output probability. The shifted probability $p_{m}$ is given by $p_{m}=max\left(p-m,0\right)$ , where m ≥ 0 is a tunable hyperparameter known as the probability margin. ASL allows the implementation of two types of asymmetry to minimize the influence of basic negative samples on the loss function. These types involve employing soft thresholding, achieved by using focusing parameters $\gamma_{-}$ > $\gamma_{+}$ , and implementing hard thresholding through the probability margin m . The total classification loss for multilabel classification is then formulated by summing the ASLs across all labels. In order to rectify the data imbalance among subclasses, we adopted single-label asymmetric loss, a modification derived from the previous multilabel asymmetric loss: the replacement of the sigmoid activation function with softmax and the exclusion of the probability margin m .

During the training phase, images were augmented with various transformations, including random horizontal and vertical flips, random rotation, color jitter, and random resized crop. The augmented images were then converted to tensors and normalized with the pre-established mean and standard deviation values from the ImageNet [43] dataset. The entire train dataset was employed to calculate the multilabel asymmetric loss for primary classes. Instances lacking precise subclass information were selectively masked for the computation of single-label loss. This loss was separately determined for DR, RVO, and AMD. The training process was governed by the aggregate loss, encompassing the sum of the multilabel ASL and the three individual single-label ASLs (Figure 1). During the evaluation phase, we analyzed performance metrics, including the area under the receiver operating characteristic curve (AUC), accuracy, recall (sensitivity), precision, F1 score (the harmonic mean of recall and precision), and specificity. To address label and subclass imbalances, we also formulated weighted variants of recall, precision, and the F1 score. Subset accuracy was also analyzed to measure the scale of samples having identical labels between the prediction and the ground-truth labels for the primary classes. In addition, we calculated Cohen’s kappa [44], specifically employing the quadratic weighted variant, to provide a comprehensive evaluation of the model’s agreement with the true labels.

All models were implemented using PyTorch framework in Python 3.10 and executed on an NVIDIA Tesla V100 SXM2 (Santa Clara, CA, USA) graphics processing unit (GPU) with 32 GB of memory. The driver version used was 510.47.03, and the CUDA version was 11.6. Throughout the training process, the random seed for shuffling data was 325, the parameter values of ASL were set as follows: a negative gamma ( $\gamma_{-}$ ) of 2, a positive gamma ( $\gamma_{+}$ ) of 1, and a probability margin ( m ) of 0.05 for multilabel ASL and a negative gamma ( $\gamma_{-}$ ) of 2 and a positive gamma ( $\gamma_{+}$ ) of 0 for single-label ASL. The training configuration comprised 3 layers and 8 heads for the encoder, a batch size of 64, a learning rate of $5\times 10^{-5}$ , a training duration spanning 100 epochs, without early stopping, and the utilization of the mean Cohen’s kappa as the metric under monitoring.

## 3. Results

### 3.1. Data Characteristics

The whole dataset was divided into three sets for developing and training the models, with a random seed of 813 to ensure replicability: the training set, consisting of 9656 images (64%); the validation set, with 2414 images (16%); and the test set, comprising 3019 images (20%) (Table 1).

### 3.2. Performance of the Models

#### 3.2.1. Classification Performance for the Primary Classes

**Figure 2:**
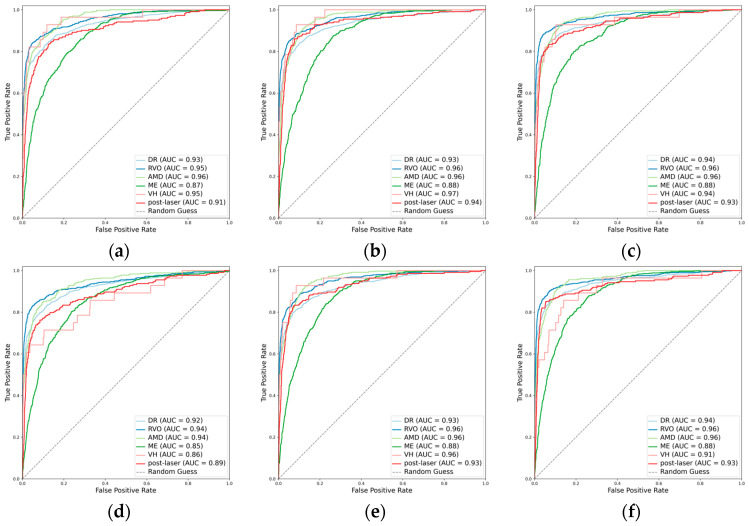
Receiver operating characteristic curves (ROCs) for the primary classes. (**a**) ResNet101, (**b**) EfficientNetV2-M, (**c**) ConvNeXt-base, (**d**) ResNet101 + attention, (**e**) EfficientNetV2-M + attention, and (**f**) ConvNeXt-base + attention.

**Figure 3:**
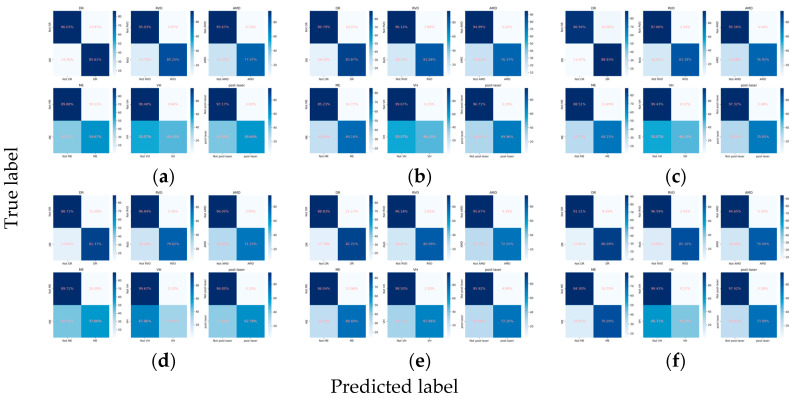
Confusion matrices for the primary classes. (**a**) ResNet101, (**b**) EfficientNetV2-M, (**c**) ConvNeXt-base, (**d**) ResNet101 + attention, (**e**) EfficientNetV2-M + attention, and (**f**) ConvNeXt-base + attention.

The integration of the attention mechanism did not enhance the overall performance of three CNN architectures for assessing the presence of the primary classes. Among these models, ConvNeXt-base emerged as the top performer, as evidenced by achieving the highest mean Cohen’s kappa of 0.653 for the primary classes, coupled with a subset accuracy of 0.644 (Table 2, Figure 2 and Figure 3).

When detecting DR, the inclusion of the attention mechanism enhanced the performance of ResNet101 (Cohen’s kappa: 0.713 vs. 0.712) and ConvNeXt-base (Cohen’s kappa: 0.778 vs. 0.751). The optimal model was identified as ConvNeXt-base + attention, attaining an AUC of 0.943 (Figure 2), an F1 score of 0.870, and a Cohen’s kappa of 0.778. For the detection of RVO, the incorporation of the attention mechanism improved the performance of ResNet101 (Cohen’s kappa: 0.786 vs. 0.778), with the pre-eminent model being ConvNeXt-base, achieving an AUC of 0.965, an F1 score of 0.863, and a Cohen’s kappa of 0.832. However, in the case of AMD, the integration did not enhance the performance of any of the three CNN architectures. The optimal model for AMD detection was ResNet101, demonstrating an AUC of 0.960, an F1 score of 0.741, and a Cohen’s kappa of 0.704. Concerning ME, the incorporation of the attention mechanism improved the performance of EfficientNetV2-M (Cohen’s kappa: 0.488 vs. 0.477), with ConvNeXt-base emerging as the superior model, achieving an AUC of 0.880, an F1 score of 0.620, and a Cohen’s kappa of 0.526. All models fell short in detecting VH (Figure 2 and Figure 3). For the identification of laser spots, the integration of the attention mechanism enhanced the performance of ResNet101 (Cohen’s kappa: 0.590 vs. 0.581) and ConvNeXt-base (Cohen’s kappa: 0.694 vs. 0.668). The optimal model was ConvNeXt-base + attention, achieving an AUC of 0.928, an F1 score of 0.717, and a Cohen’s kappa of 0.694 (Table 2).

#### 3.2.2. Classification Performance for the DR Subclasses

**Figure 4:**
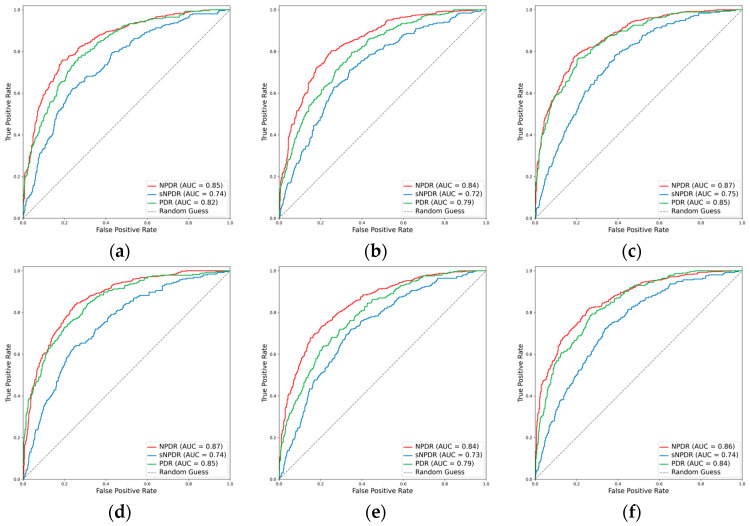
ROCs for the DR subclasses. (**a**) ResNet101, (**b**) EfficientNetV2-M, (**c**) ConvNeXt-base, (**d**) ResNet101 + attention, (**e**) EfficientNetV2-M + attention, and (**f**) ConvNeXt-base + attention.

**Figure 5:**
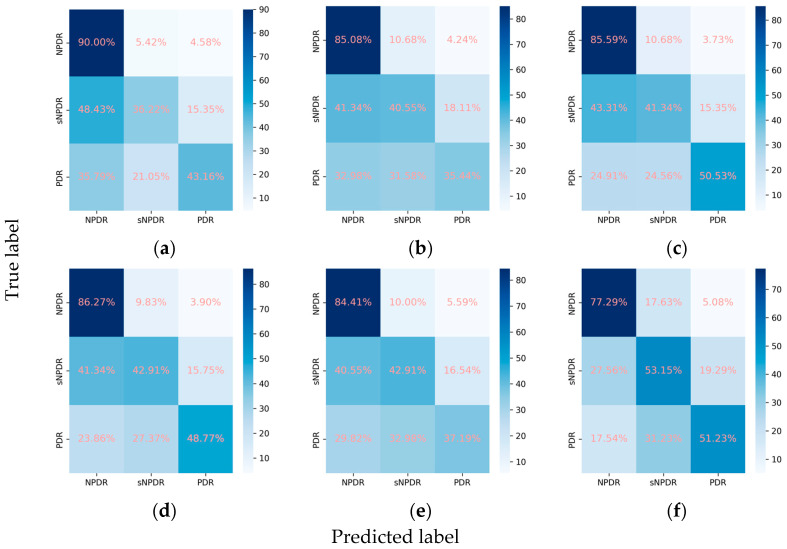
Confusion matrices for the DR subclasses. (**a**) ResNet101, (**b**) EfficientNetV2-M, (**c**) ConvNeXt-base, (**d**) ResNet101 + attention, (**e**) EfficientNetV2-M + attention, and (**f**) ConvNeXt-base + attention.

Despite the suboptimal performance of all the models in accurately grading DR into NPDR, sNPDR, and PDR, the incorporation of the attention mechanism led to an overall enhancement in the performance metrics. Specifically, for ResNet101, there was an improvement in the weighted F1 score from 0.638 to 0.661 and in Cohen’s kappa from 0.491 to 0.570. Similarly, EfficientNetV2-M demonstrated an increase in the weighted F1 score from 0.609 to 0.618 and in Cohen’s kappa from 0.467 to 0.476, while ConvNeXt-base exhibited an improvement in Cohen’s kappa from 0.566 to 0.575. Among these models, ConvNeXt-base + attention emerged as the optimal model (Figure 4 and Figure 5). This model achieved a weighted F1 score of 0.658 and a Cohen’s kappa of 0.575, showcasing superior performance in grading the DR severity (Table 2, Figure 4 and Figure 5).

#### 3.2.3. Classification Performance for the RVO Subclasses

**Figure 6:**
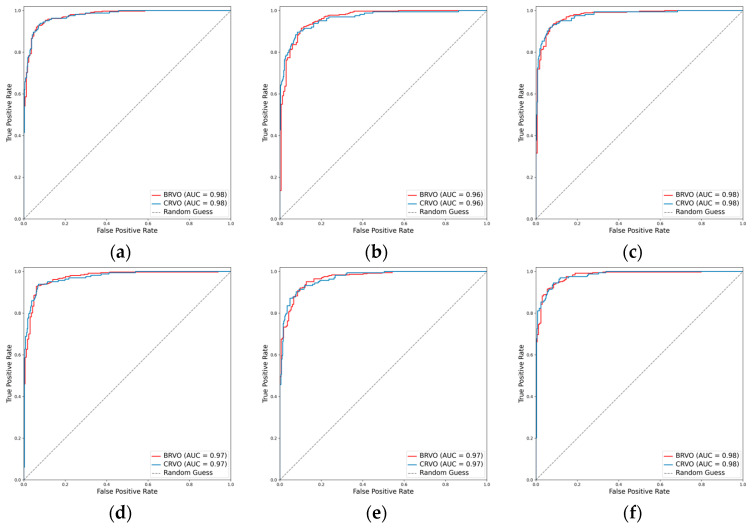
ROCs for the RVO subclasses. (**a**) ResNet101, (**b**) EfficientNetV2-M, (**c**) ConvNeXt-base, (**d**) ResNet101 + attention, (**e**) EfficientNetV2-M + attention, and (**f**) ConvNeXt-base + attention.

**Figure 7:**
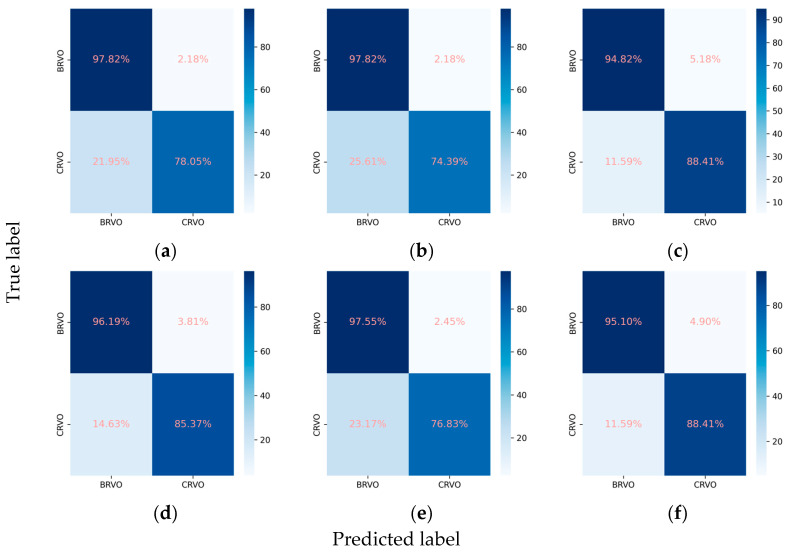
Confusion matrices for the RVO subclasses. (**a**) ResNet101, (**b**) EfficientNetV2-M, (**c**) ConvNeXt-base, (**d**) ResNet101 + attention, (**e**) EfficientNetV2-M + attention, and (**f**) ConvNeXt-base + attention.

For the classification of RVO into BRVO and CRVO, the incorporation of the attention mechanism enhanced the performance of all three CNNs. In the case of ResNet101, the weighted F1 score increased from 0.915 to 0.928, and Cohen’s kappa increased from 0.796 to 0.829. Similarly, EfficientNetV2-M exhibited an increase in the weighted F1 score from 0.902 to 0.909 and in Cohen’s kappa from 0.766 to 0.782. Concurrently, ConvNeXt-base demonstrated an improvement in the weighted F1 score from 0.928 to 0.930 and in Cohen’s kappa from 0.832 to 0.837. Among these models, ConvNeXt-base + attention emerged as the optimal model, achieving an AUC of 0.981, a weighted F1 score of 0.930, and a Cohen’s kappa of 0.837 (Table 2, Figure 6 and Figure 7).

#### 3.2.4. Classification Performance for the AMD Subclasses

**Figure 8:**
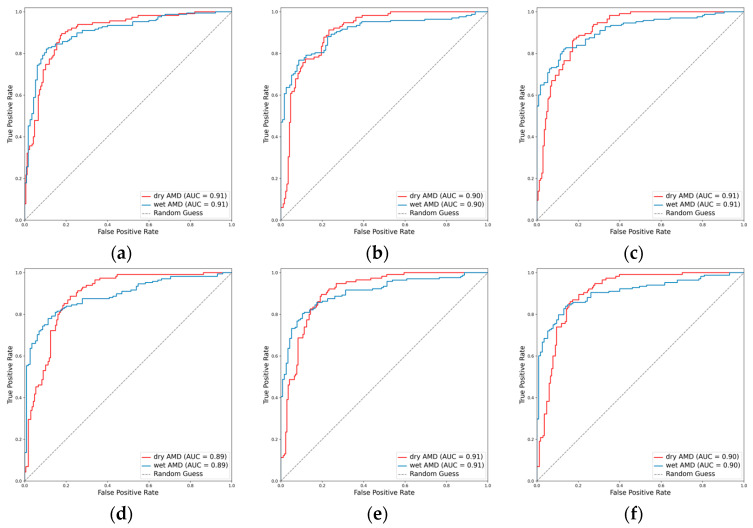
ROCs for the AMD subclasses. (**a**) ResNet101, (**b**) EfficientNetV2-M, (**c**) ConvNeXt-base, (**d**) ResNet101 + attention, (**e**) EfficientNetV2-M + attention, and (**f**) ConvNeXt-base + attention.

**Figure 9:**
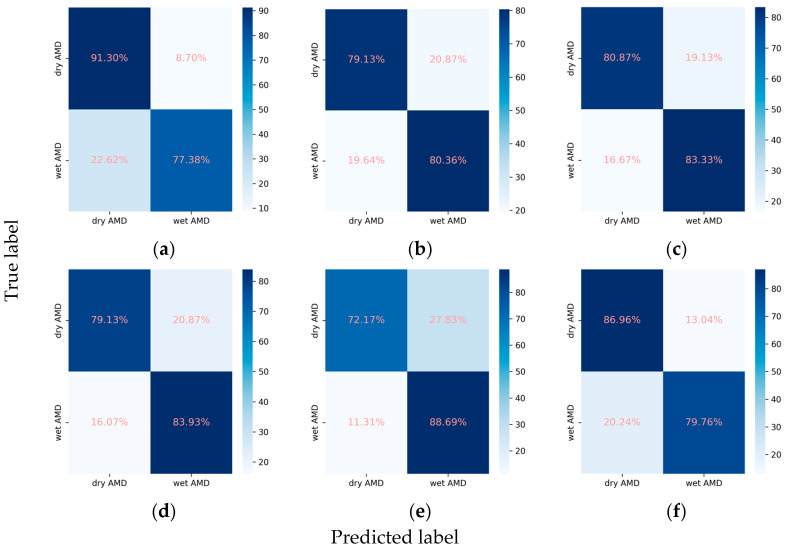
Confusion matrices for the AMD subclasses. (**a**) ResNet101, (**b**) EfficientNetV2-M, (**c**) ConvNeXt-base, (**d**) ResNet101 + attention, (**e**) EfficientNetV2-M + attention, and (**f**) ConvNeXt-base + attention.

In the task of classifying AMD into dry and wet types, the integration of the attention mechanism proved beneficial for the performance of EfficientNetV2-M and ConvNeXt-base. Specifically, EfficientNetV2-M demonstrated an increase in the weighted F1 score from 0.800 to 0.818 and in Cohen’s kappa from 0.588 to 0.620. Similarly, ConvNeXt-base exhibited improvement in the weighted F1 score from 0.824 to 0.828 and in Cohen’s kappa from 0.637 to 0.650. Among these models, ResNet101 emerged as the optimal, achieving an AUC of 0.906, a weighted F1 score of 0.832, and a Cohen’s kappa of 0.661 (Table 2, Figure 8 and Figure 9).

### 3.3. Ablation Studies of Key Parameters

Although ConvNeXt-base stood out as the top performer, achieving the highest mean Cohen’s kappa of 0.661, the ConvNeXt-base + attention model excelled in accurately classifying the subclasses for DR, RVO, and AMD while maintaining precision in assessing the primary classes. To assess the impact of key parameters, including the negative gamma ( $\gamma_{-}$ ) and the number of encoder layers and heads, on the ConvNeXt-base + attention model, we conducted ablation studies. The experiments involved varying the negative gamma to values of 3 and 4, setting the number of encoder layers to 4 and 5 while keeping the number of heads fixed at 8, and adjusting the number of heads to 16 and 32 with the number of layers fixed at 3. Other parameters were held constant throughout these experiments.

The augmentation of the negative gamma resulted in a decline in the model’s performance. Specifically, the mean Cohen’s kappa decreased from 0.660 to 0.645 and further to 0.640 (Supplementary Table S1). This deterioration underscores the sensitivity of the model to changes in the negative gamma parameter. Increasing the number of encoder layers, on the other hand, had a positive impact on performance, with the mean Cohen’s kappa rising from 0.660 to 0.666 (Supplementary Table S2). Notably, this enhancement was particularly prominent in predicting the presence of the primary classes. The mean Cohen’s kappa for the primary classes increased from 0.646 to 0.662 and further to 0.670 (Supplementary Table S2). However, there was no significant increase in Cohen’s kappa for the classification of the DR, RVO, or AMD subclasses (Supplementary Table S2). Among the three options for the number of encoder heads, 16 proved to be the most effective, resulting in the highest mean Cohen’s kappa of 0.667. Nevertheless, the improvement was more noticeable for the primary classes, whereas there was not a comparable enhancement in the classification of the DR, RVO, or AMD subclasses (Supplementary Table S3).

## 4. Discussion 

In a groundbreaking study, Gulshan et al. utilized a dataset of 128,175 retinal images to develop a deep learning (DL) algorithm. This algorithm exhibited an exceptional ability to identify moderate or worse DR, boasting a sensitivity and specificity exceeding 90% [15]. These findings were corroborated by Ting et al., who demonstrated the DL algorithm’s comparable efficacy in detecting various ocular conditions such as possible glaucoma and AMD [16]. Chen et al. employed DL models to screen for RVO [27]. Their investigation revealed that the Inception-v3 model exhibited notable performance, achieving a sensitivity of 0.93, specificity of 0.99, and an F1 score of 0.95 in RVO identification. Similarly, Ren et al. achieved remarkable outcomes with their RVO identification model. In an independent dataset, their model exhibited robust performance, maintaining an impressive AUC of 0.81 and accurately detecting RVO [28].

Our innovative approach distinguishes itself from conventional models that typically specialize in a singular task, such as grading DR or classifying RVO or AMD. Through the strategic integration of an attention mechanism and the implementation of a hierarchical multilabel classification task design, our models exhibited a remarkable ability to initially assess the presence of DR, RVO, AMD, ME, and laser spots with commendable recall and specificity. Subsequently, the models proceeded to classify subclasses of DR, RVO, and AMD if the output probability exceeded or equaled 0.5.

**Figure 10:**
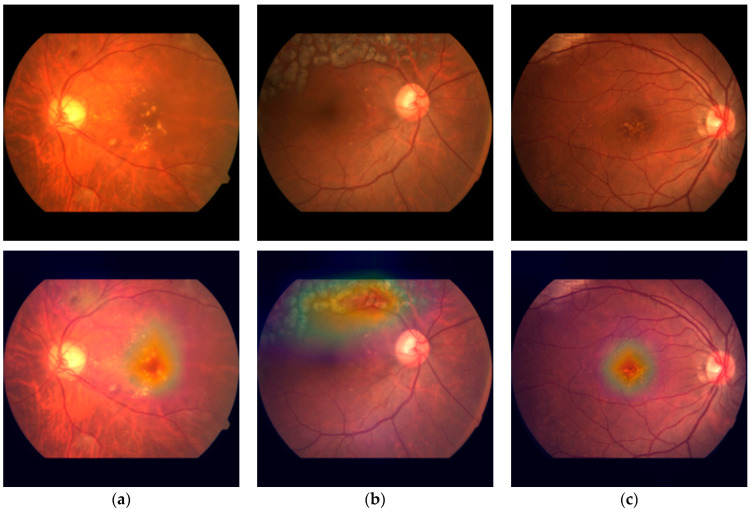
Visualization of the attention weights of the ConvNext-base + attention model in CFPs. (**a**) A case of DR, ME, and sNPDR, (**b**) a case of RVO, laser spots, and BRVO, and (**c**) a case of AMD and dry AMD.

Our methodology places a significant emphasis on the interpretability of DL models, a critical element in comprehending their decision-making processes. We integrated an attention mechanism, initially devised for natural language processing tasks [37], to enable our models to calculate global relative scores across features. This integration proved to be pivotal in elevating the performance of our classification models, particularly the ConvNeXt + attention model, which demonstrated accurate subclass classification for DR, RVO, and AMD while maintaining the precision for assessing the primary classes. Moreover, this incorporation did not impose a significant time complexity burden on the models. The additional time required for training one epoch was merely a matter of seconds. In order to offer visual insights into the decision-making process, we effectively employed heatmaps to depict attention weights in CFPs (Figure 10). This approach not only enhances the transparency of our models but also provides a valuable tool for clinicians and researchers, enabling them to grasp the rationale behind the model’s decisions.

Despite these promising results, challenges persist in accurately identifying VH, sNPDR and PDR, as indicated by suboptimal sensitivity and F1 score. This limitation can be ascribed to various factors, encompassing the scarcity of VH occurrences within the datasets, the intricate challenges posed by the indistinguishable similarities between sNPDR and NPDR or PDR, and the inherent limitations arising from the reliance solely on CFPs. Additionally, the diagnoses were derived from FFA images, leading to potential gaps in the available information. To delve deeper into addressing these challenges, exploring advanced techniques such as transfer learning or ensemble models could be beneficial. Transfer learning involves leveraging pretrained models on large datasets and fine-tuning them for the specific task at hand, potentially mitigating the impact of data imbalance [45]. Ensemble models, on the other hand, combine predictions from multiple models, often enhancing overall performance and robustness [46]. Additionally, incorporating multimodal data, such as patient clinical records, optical coherence tomography angiography (OCTA) scans [47,48] or FFA images when deemed necessary, may provide a more comprehensive perspective of the disease, potentially improving the accuracy of diagnosis. This integration of diverse data modalities can offer complementary information, helping to overcome limitations associated with a single-source data approach. This remains a focal point of our ongoing efforts as we strive to advance our work in the future. Additionally, the study’s scope was limited to a single ophthalmology center, where varying diagnostic accuracy among contributing doctors impacted the consistency of interpretation. Future studies could benefit from a more diverse and extensive dataset, potentially addressing these limitations for more comprehensive and accurate results.

## Figures and Tables

**Table 1:** Patient demographics and characteristics in the datasets.

Characteristic		Datasets			
		Train	Validate	Test	Whole
Number of images (eyes)		9656	2414	3019	15,089
Number of unique patients ^a^		6768	2245	2719	8110
Age, mean (SD ^b^) ^c^		66.40 (14.35)	66.24 (14.50)	66.30 (14.17)	66.40 (14.35)
Sex, female (%) ^c^		3317 (49.01)	1093 (45.28)	1342 (49.36)	3963 (48.87)
**Diagnoses ^c^**					
Primary Classes	DR	3973	953	1265	6191
	RVO	1848	477	584	2909
	AMD	1131	314	364	1809
	ME	1775	454	548	2777
	VH	111	28	28	167
	Laser spots	759	199	223	1181
DR subclasses	NPDR	1820	422	590	2832
	sNPDR	777	201	254	1232
	PDR	928	223	285	1436
	Unspecified	448	107	136	691
RVO subclasses	BRVO	1081	290	367	1738
	CRVO	580	147	164	891
	Unspecified	187	40	53	280
AMD subclasses	Dry AMD	328	105	115	548
	Wet AMD	507	127	168	802
	Unspecified	296	82	81	459

^a^ The whole dataset is divided based on eye side, where the train, validate, and test datasets may share patients, but each eye is unique and not shared across the splits. ^b^ SD, standard deviation. ^c^ Age, sex, and diagnoses have no significant differences among the four datasets. The ANOVA *p*-value for age is 0.95, the chi-square *p*-value for sex is 0.96, and the chi-square *p*-value for diagnoses is 0.98.

**Table 2:** Performance of the models.

Diagnoses/Metrics		Models	AUC	Accuracy	Recall	Precision	F1 Score	Specificity	Cohen’s Kappa
Primary classes	DR	R	0.930	0.859	0.856	0.816	0.835	0.860	0.712
		R + A	0.923	0.861	0.824	0.840	0.832	0.887	0.713
		E	0.933	0.873	0.839	0.856	0.847	0.898	0.739
		E + A	0.929	0.861	0.822	0.841	0.832	0.888	0.713
		C	0.942	0.878	**0.889**	0.831	0.859	0.869	0.751
		C + A	**0.943**	**0.892**	0.861	**0.880**	**0.870**	**0.915**	**0.778**
	RVO	R	0.954	0.929	0.842	0.803	0.822	0.950	0.778
		R + A	0.939	0.935	0.796	0.858	0.826	0.968	0.786
		E	0.958	0.933	0.817	0.835	0.826	0.961	0.785
		E + A	0.955	0.932	0.810	0.836	0.823	0.962	0.781
		C	**0.965**	**0.949**	0.834	**0.895**	**0.863**	**0.977**	**0.832**
		C + A	0.960	0.944	**0.851**	0.857	0.854	0.966	0.819
	AMD	R	0.960	**0.935**	0.775	0.710	**0.741**	0.957	**0.704**
		R + A	0.941	0.930	0.712	**0.712**	0.712	**0.960**	0.672
		E	0.958	0.927	0.764	0.676	0.717	0.950	0.676
		E + A	0.960	0.928	0.723	0.696	0.709	0.957	0.668
		C	**0.962**	0.933	0.769	0.704	0.735	0.956	0.697
		C + A	0.959	0.928	**0.794**	0.671	0.727	0.947	0.686
	ME	R	0.874	0.844	0.597	0.567	0.581	0.899	0.486
		R + A	0.851	0.839	0.577	0.554	0.565	0.897	0.467
		E	0.877	0.823	0.692	0.509	0.587	0.852	0.477
		E + A	0.881	0.829	0.688	0.522	0.594	0.860	0.488
		C	0.880	**0.848**	0.682	**0.568**	**0.620**	0.885	**0.526**
		C + A	**0.885**	0.828	**0.761**	0.518	0.616	0.843	0.511
	VH	R	0.955	0.989	0.464	0.419	0.441	0.994	0.435
		R + A	0.857	0.990	0.321	0.474	0.383	**0.997**	0.378
		E	**0.969**	**0.992**	0.464	**0.565**	**0.510**	**0.997**	**0.506**
		E + A	0.956	0.982	**0.679**	0.297	0.413	0.985	0.405
		C	0.943	0.989	0.464	0.433	0.448	0.994	0.443
		C + A	0.911	0.989	0.393	0.393	0.393	0.994	0.387
	Laser spots	R	0.906	0.944	0.596	0.627	0.611	0.972	0.581
		R + A	0.888	0.943	0.628	0.614	0.621	0.969	0.590
		E	**0.935**	0.947	0.700	0.629	0.662	0.967	0.634
		E + A	0.928	0.942	0.722	0.585	0.647	0.959	0.615
		C	0.932	0.954	0.709	0.678	0.693	0.973	0.668
		C + A	0.928	**0.957**	**0.740**	**0.696**	**0.717**	**0.974**	**0.694**
Weighted metrics and mean Cohen’s kappa for the primary classes		R	-	-	0.774	0.737	0.755	-	0.616
		R + A	-	-	0.741	0.756	0.748	-	0.601
		E	-	-	0.785	0.748	0.763	-	0.636
		E + A	-	-	0.775	0.741	0.754	-	0.612
		C	-	-	0.809	0.765	0.785	-	**0.653**
		C + A	-	-	**0.819**	**0.766**	**0.788**	-	0.646
Subset accuracy		R	-	0.610	-	-	-	-	-
		R + A	-	0.604	-	-	-	-	-
		E	-	0.600	-	-	-	-	-
		E + A	-	0.589	-	-	-	-	-
		C	-	**0.644**	-	-	-	-	-
		C + A	-	0.629	-	-	-	-	-
DR subclasses	NPDR	R	0.850	0.748	**0.900**	0.702	0.789	0.583	-
		R + A	**0.865**	0.775	0.863	0.746	**0.800**	0.679	-
		E	0.843	0.746	0.851	0.716	0.778	0.631	-
		E + A	0.839	0.752	0.844	0.726	0.781	0.651	-
		C	**0.865**	0.764	0.855	0.736	0.792	0.664	-
		C + A	0.859	**0.775**	0.773	**0.792**	0.782	**0.777**	-
	sNPDR	R	0.741	**0.775**	0.362	**0.500**	0.420	**0.895**	-
		R + A	0.743	0.751	0.429	0.445	0.437	0.845	-
		E	0.725	0.731	0.406	0.402	0.404	0.825	-
		E + A	0.725	0.736	0.429	0.416	0.422	0.825	-
		C	**0.751**	0.750	0.413	0.441	0.427	0.848	-
		C + A	0.741	0.724	**0.531**	0.412	**0.464**	0.779	-
	PDR	R	0.823	0.798	0.432	0.651	0.519	0.922	-
		R + A	0.849	0.815	0.488	0.688	0.571	0.925	-
		E	0.794	0.774	0.354	0.587	0.442	0.916	-
		E + A	0.789	0.775	0.372	0.587	0.455	0.911	-
		C	**0.853**	**0.821**	0.505	**0.702**	**0.588**	**0.928**	-
		C + A	0.837	0.807	**0.512**	0.649	0.573	0.906	-
Weighted metrics and Cohen’s kappa for the DR subclasses		R	-	-	0.661	0.644	0.638	-	0.491
		R + A	-	-	**0.671**	0.664	**0.661**	-	0.570
		E	-	-	0.625	0.613	0.609	-	0.467
		E + A	-	-	0.632	0.621	0.618	-	0.476
		C	-	-	0.668	0.661	0.658	-	0.566
		C + A	-	-	0.653	**0.670**	0.658	-	**0.575**
RVO subclasses	BRVO	R	0.976	0.917	**0.978**	0.909	0.942	0.780	-
		R + A	0.972	0.928	0.962	0.936	0.949	0.854	-
		E	0.963	0.906	**0.978**	0.895	0.935	0.744	-
		E + A	0.969	0.911	0.975	0.904	0.938	0.768	-
		C	0.976	0.928	0.948	**0.948**	0.948	**0.884**	-
		C + A	**0.981**	**0.930**	0.951	**0.948**	**0.950**	**0.884**	-
	CRVO	R	0.976	0.917	0.780	**0.941**	0.853	**0.978**	-
		R + A	0.972	0.928	0.854	0.909	0.881	0.962	-
		E	0.963	0.906	0.744	0.938	0.830	**0.978**	-
		E + A	0.969	0.911	0.768	0.933	0.843	0.975	-
		C	0.976	0.928	**0.884**	0.884	0.884	0.948	-
		C + A	**0.981**	**0.930**	**0.884**	0.890	**0.887**	0.951	-
Weighted metrics and Cohen’s kappa for the RVO subclasses		R	-	-	0.917	0.919	0.915	-	0.796
		R + A	-	-	0.928	0.928	0.928	-	0.829
		E	-	-	0.906	0.907	0.902	-	0.766
		E + A	-	-	0.911	0.913	0.909	-	0.782
		C	-	-	0.928	0.928	0.928	-	0.832
		C + A	-	-	**0.930**	**0.930**	**0.930**	**-**	**0.837**
AMD subclasses	Dry AMD	R	0.906	**0.830**	**0.913**	0.734	**0.814**	0.774	-
		R + A	0.890	0.820	0.791	0.771	0.781	0.839	-
		E	0.905	0.799	0.791	0.734	0.762	0.804	-
		E + A	0.907	0.820	0.722	**0.814**	0.765	**0.887**	-
		C	**0.912**	0.823	0.809	0.769	0.788	0.833	-
		C + A	0.905	0.827	0.870	0.746	0.803	0.798	-
	Wet AMD	R	0.906	**0.830**	0.774	**0.926**	0.844	**0.913**	-
		R + A	0.890	0.820	0.839	0.855	0.847	0.791	-
		E	0.905	0.799	0.804	0.849	0.826	0.791	-
		E + A	0.907	0.820	**0.887**	0.823	**0.854**	0.722	-
		C	**0.912**	0.823	0.833	0.864	0.848	0.809	-
		C + A	0.905	0.827	0.798	0.899	0.845	0.870	-
Weighted metrics and Cohen’s kappa for the AMD subclasses		R	-	-	**0.830**	**0.850**	**0.832**	**-**	**0.661**
		R + A	-	-	0.820	0.821	0.820	-	0.628
		E	-	-	0.799	0.802	0.800	-	0.588
		E + A	-	-	0.820	0.820	0.818	-	0.620
		C	-	-	0.823	0.825	0.824	-	0.637
		C + A	-	-	0.827	0.837	0.828	-	0.650
Mean Cohen’s kappa (best epoch/100)		R	-	-	-	-	-	-	0.627 (16)
		R + A	-	-	-	-	-	-	0.626 (73)
		E	-	-	-	-	-	-	0.626 (12)
		E + A	-	-	-	-	-	-	0.616 (9)
		C	-	-	-	-	-	-	**0.661** (24)
		C + A	-	-	-	-	-	-	0.660 (27)

Best values are highlighted in bold. R, ResNet101; E, EfficientNetV2-M; C, ConvNeXt-base; and A, attention.

## Data Availability

The data that support the findings of this study are available from the corresponding author upon reasonable request.

## Supplementary Materials

The following supporting information can be downloaded at: https://www.mdpi.com/2075-4418/14/2/121/s1. Table S1. Ablation studies on negative gamma ( $\gamma_{-}$ ). Table S2. Ablation studies on the number of layers. Table S3. Ablation studies on the number of heads.
