# Supplementary material for: Interpretable Detection of Diabetic Retinopathy, Retinal Vein Occlusion, Age-Related Macular Degeneration, and Other Fundus Conditions
Source: Diagnostics (Basel). 2024 Jan 5;14(2):121. doi: 10.3390/diagnostics14020121 (PMC11487407; doi:10.3390/diagnostics14020121)
Supplement: Supplementary file 1 [file diagnostics-14-00121-s001.zip › diagnostics-2735608-supplementary.pdf]

Interpretable Detection of Diabetic Retinopathy, Retinal Vein Occlusion, Age-related Macular Degeneration and Other Fundus Conditions

Wenlong Li[1,2], Linbo Bian[1,2], Baikai Ma[1,2], Tong Sun[1,2], Yiyun Liu[1,2], Zhengze Sun[1,2], Lin Zhao[1,2], Kang Feng[1,2], Fan Yang[1,2], Xiaona Wang[1,2], Szyyann Chan[1,2], Hongliang Dou[1,2,*], Hong Qi[1,2,*]

[1] Department of Ophthalmology, Peking University Third Hospital, Beijing 100191, China;

[2] Beijing Key Laboratory of Restoration of Damaged Ocular Nerve, Beijing 100191, China

[*] Correspondence: douhl@aliyun.com (H.D.); doctorqihong@163.com (H.Q.)

Supplementary Table S1. Ablation studies on negative gamma (γ_-)

Diagnoses/ Metrics		γ_-	AUC	Accuracy	Recall	Precision	F1-score	Specificity	Cohen's kappa
Primary Classes	DR	2	0.943	**0.892**	0.861	**0.880**	**0.870**	**0.915**	**0.778**
		3	**0.947**	0.886	0.866	0.862	0.864	0.900	0.765
		4	0.937	0.875	**0.869**	0.839	0.854	0.880	0.745
	RVO	2	0.960	**0.944**	0.851	**0.857**	0.854	**0.966**	0.819
		3	**0.966**	0.943	**0.861**	0.848	**0.855**	0.963	**0.820**
		4	0.958	0.941	0.856	0.843	0.850	0.962	0.813
	AMD	2	0.959	0.928	0.794	0.671	0.727	0.947	0.686
		3	**0.966**	0.925	**0.863**	0.641	**0.735**	0.934	**0.693**
		4	0.953	**0.930**	0.745	**0.698**	0.721	**0.956**	0.681
	ME	2	0.885	0.828	**0.761**	0.518	**0.616**	0.843	**0.511**
		3	**0.892**	0.830	0.737	0.523	0.612	0.851	0.508
		4	0.857	**0.836**	0.630	**0.541**	0.582	**0.881**	0.480
	VH	2	0.911	**0.989**	**0.393**	**0.393**	**0.393**	0.994	**0.387**
		3	**0.944**	**0.989**	0.286	0.381	0.327	**0.996**	0.321
		4	0.911	0.987	0.357	0.333	0.345	0.993	0.338
	Laser spots	2	0.928	**0.957**	0.740	**0.696**	0.717	**0.974**	0.694
		3	**0.936**	**0.957**	**0.767**	0.687	**0.725**	0.972	**0.701**
		4	0.913	0.952	0.726	0.656	0.689	0.970	0.663
Weighted metrics and mean Cohen's kappa for the primary classes		2	-	-	0.819	**0.766**	**0.788**	-	**0.646**
		3	-	-	**0.828**	0.754	0.785	-	0.635
		4	-	-	0.792	0.750	0.770	-	0.620
Subset accuracy		2	-	**0.629**	-	-	-	-	-
		3	-	0.628	-	-	-	-	-
		4	-	0.615	-	-	-	-	-
DR subclasses	NPDR	2	0.859	**0.775**	0.773	**0.792**	0.782	**0.777**	-
		3	0.866	0.764	**0.885**	0.725	**0.797**	0.633	-
		4	**0.869**	0.764	0.844	0.740	0.789	0.675	-
	sNPDR	2	0.741	0.724	**0.531**	0.412	**0.464**	0.779	-
		3	0.746	0.758	0.386	0.456	0.418	0.866	-
		4	**0.755**	**0.760**	0.354	**0.457**	0.399	**0.878**	-
	PDR	2	0.837	0.807	0.512	0.649	0.573	**0.906**	-
		3	0.841	0.804	0.453	**0.665**	0.539	0.923	-
		4	**0.861**	**0.816**	**0.589**	0.649	**0.618**	0.892	-
Weighted metrics and Cohen's kappa for the DR subclasses		2	-	-	0.653	**0.670**	**0.658**	-	**0.575**
		3	-	-	0.663	0.649	0.646	-	0.534
		4	-	-	**0.670**	0.653	**0.658**	-	0.571
RVO subclasses	BRVO	2	**0.981**	0.930	0.951	**0.948**	0.950	**0.884**	-
		3	0.980	0.923	**0.986**	0.910	0.946	0.780	-
		4	0.977	**0.937**	0.970	0.942	**0.956**	0.866	-

	CRVO	2	**0.981**	0.930	**0.884**	0.890	0.887	0.951	-
		3	0.980	0.923	0.780	**0.962**	0.862	**0.986**	-
		4	0.977	**0.938**	0.866	0.928	**0.896**	0.970	-
Weighted metrics and Cohen's kappa for the RVO subclasses		2	-	-	0.930	0.930	0.930	-	0.837
		3	-	-	0.923	0.926	0.920	-	0.809
		4	-	-	**0.938**	**0.938**	**0.937**	-	**0.852**
AMD subclasses	Dry AMD	2	**0.905**	**0.827**	0.870	**0.746**	0.803	**0.798**	-
		3	0.896	**0.827**	**0.878**	0.743	**0.805**	0.792	-
		4	0.883	0.809	0.861	0.723	0.786	0.774	-
	Wet AMD	2	**0.905**	**0.827**	**0.798**	0.899	**0.845**	0.870	-
		3	0.896	**0.827**	0.792	**0.905**	0.844	**0.878**	-
		4	0.883	0.809	0.774	0.890	0.828	0.861	-
Weighted metrics and Cohen's kappa for the AMD subclasses		2	-	-	**0.827**	0.837	**0.828**		0.650
		3	-	-	**0.827**	**0.839**	**0.828**		**0.651**
		4	-	-	0.809	0.822	0.811		0.616
Mean Cohen's kappa (best epoch/100)		2	-	-	-	-	-	-	**0.660** (27)
		3	-	-	-	-	-	-	0.645 (22)
		4	-	-	-	-	-	-	0.640 (86)

Best values are highlighted in bold.

Supplementary Table S2. Ablation studies on the number of layers

Diagnoses/ Metrics		Number of layers	AUC	Accuracy	Recall	Precision	F1-score	Specificity	Cohen's kappa
Primary Classes	DR	3	0.943	0.892	**0.861**	0.880	**0.870**	0.915	0.778
		4	**0.948**	0.893	0.847	0.893	0.869	0.926	0.779
		5	**0.948**	**0.895**	0.832	**0.909**	0.869	**0.940**	**0.781**
	RVO	3	0.960	0.944	0.851	**0.857**	0.854	**0.966**	0.819
		4	**0.972**	0.941	0.853	0.843	0.848	0.962	0.811
		5	0.969	**0.947**	**0.877**	0.853	**0.865**	0.964	**0.832**
	AMD	3	0.959	0.928	0.794	0.671	0.727	0.947	0.686
		4	**0.967**	**0.938**	0.788	**0.721**	**0.753**	**0.958**	**0.718**
		5	0.965	0.930	**0.819**	0.673	0.739	0.945	0.699
	ME	3	0.885	0.828	**0.761**	0.518	**0.616**	0.843	**0.511**
		4	**0.896**	0.833	0.719	0.530	0.610	0.859	0.507
		5	0.891	**0.840**	0.682	**0.548**	0.608	**0.875**	0.509
	VH	3	0.911	0.989	0.393	0.393	0.393	0.994	0.387
		4	0.956	**0.991**	0.429	**0.500**	0.462	**0.996**	0.457
		5	**0.968**	**0.991**	**0.500**	**0.500**	**0.500**	0.995	**0.495**
	Laser spots	3	0.928	0.957	0.740	0.696	0.717	0.974	0.694
		4	**0.942**	0.953	**0.821**	0.644	0.722	0.964	0.697
		5	0.941	**0.958**	0.749	**0.702**	**0.725**	**0.975**	**0.702**
Weighted metrics and mean Cohen's kappa for the primary classes		3	-	-	**0.819**	0.766	0.788	-	0.646
		4	-	-	0.812	0.774	0.789	-	0.662
		5	-	-	0.802	**0.785**	**0.791**	-	**0.670**
Subset accuracy		3	-	0.629	-	-	-	-	-
		4	-	0.645	-	-	-	-	-
		5	-	**0.648**	-	-	-	-	-
DR subclasses	NPDR	3	**0.859**	**0.775**	0.773	**0.792**	0.782	**0.777**	-
		4	0.856	0.755	0.836	0.733	0.781	0.666	-
		5	0.852	0.756	**0.869**	0.722	**0.789**	0.633	-
	sNPDR	3	**0.741**	0.724	**0.531**	0.412	**0.464**	0.779	-
		4	0.740	0.739	0.437	0.422	0.429	0.826	-
		5	0.739	**0.764**	0.291	**0.460**	0.357	**0.901**	-
	PDR	3	**0.837**	**0.807**	0.512	**0.649**	**0.573**	0.906	-
		4	0.815	0.780	0.404	0.596	0.481	**0.908**	-
		5	0.808	0.780	**0.516**	0.572	0.542	0.870	-
Weighted metrics and Cohen's kappa for the DR subclasses		3	-	-	**0.653**	**0.670**	**0.658**	-	**0.575**
		4	-	-	0.637	0.628	0.626	-	0.490
		5	-	-	0.650	0.625	0.629	-	0.512
RVO subclasses	BRVO	3	**0.981**	0.930	0.951	**0.948**	0.950	**0.884**	-
		4	0.974	0.927	**0.978**	0.921	0.948	0.811	-
		5	0.980	**0.932**	0.973	0.932	**0.952**	0.841	-

	CRVO	3	**0.981**	0.930	**0.884**	0.890	**0.887**	0.951	-
		4	0.974	0.927	0.811	**0.943**	0.872	**0.978**	-
		5	0.980	**0.932**	0.841	0.932	0.885	0.973	-
Weighted metrics and Cohen's kappa for the RVO subclasses		3	-	-	0.930	0.930	0.930	-	**0.837**
		4	-	-	0.927	0.928	0.925	-	0.821
		5	-	-	**0.932**	**0.932**	**0.931**	-	**0.837**
AMD subclasses	Dry AMD	3	0.905	0.827	0.870	**0.746**	0.803	**0.798**	-
		4	0.908	**0.830**	0.896	0.741	**0.811**	0.786	-
		5	**0.910**	0.813	**0.930**	0.704	0.801	0.732	-
	Wet AMD	3	0.905	0.827	**0.798**	0.899	0.845	0.870	-
		4	0.908	**0.830**	0.786	0.917	**0.846**	0.896	-
		5	**0.910**	0.813	0.732	**0.939**	0.823	**0.930**	-
Weighted metrics and Cohen's kappa for the AMD subclasses		3	-	-	0.827	0.837	0.828	-	0.650
		4	-	-	**0.830**	**0.845**	**0.832**	-	**0.660**
		5	-	-	0.813	0.843	0.814	-	0.631
Mean Cohen's kappa (best epoch/100)		3	-	-	-	-	-	-	0.660 (27)
		4	-	-	-	-	-	-	0.660 (15)
		5	-	-	-	-	-	-	**0.666** (16)

Best values are highlighted in bold.

Supplementary Table S3. Ablation studies on the number of heads

Diagnoses/ Metrics		Number of heads	AUC	Accuracy	Recall	Precision	F1-score	Specificity	Cohen's kappa
Primary Classes	DR	8	0.943	0.892	0.861	0.880	0.870	0.915	0.778
		16	**0.949**	0.892	**0.877**	0.868	**0.872**	0.904	**0.779**
		32	0.948	**0.893**	0.840	**0.899**	0.868	**0.932**	**0.779**
	RVO	8	0.960	0.944	0.851	**0.857**	0.854	**0.966**	0.819
		16	**0.972**	0.943	0.860	0.849	0.854	0.963	0.819
		32	0.968	**0.945**	**0.863**	0.853	**0.858**	0.964	**0.824**
	AMD	8	0.959	0.928	0.794	0.671	0.727	0.947	0.686
		16	0.966	0.918	**0.843**	0.615	0.711	0.928	0.665
		32	**0.967**	**0.934**	0.821	**0.691**	**0.750**	**0.950**	**0.713**
	ME	8	0.885	0.828	**0.761**	0.518	**0.616**	0.843	0.511
		16	**0.894**	0.835	0.724	0.533	0.614	0.859	0.512
		32	0.890	**0.843**	0.684	**0.554**	0.612	**0.878**	**0.515**
	VH	8	0.911	0.989	0.393	0.393	0.393	0.994	0.387
		16	**0.963**	**0.991**	**0.500**	**0.500**	**0.500**	**0.995**	**0.495**
		32	0.955	0.988	0.286	0.333	0.308	0.995	0.302
	Laser spots	8	0.928	0.957	0.740	**0.696**	0.717	**0.974**	0.694
		16	**0.948**	**0.958**	0.785	0.692	0.735	0.972	**0.713**
		32	0.945	0.957	**0.807**	0.677	**0.736**	0.969	**0.713**
Weighted metrics and mean Cohen's kappa for the primary classes		8	-	-	0.819	0.766	0.788	-	0.646
		16	-	-	**0.831**	0.756	0.789	-	**0.664**
		32	-	-	0.806	**0.780**	**0.790**	-	0.641
Subset accuracy [a]		8	-	0.629	-	-	-	-	-
		16	-	0.629	-	-	-	-	-
		32	-	**0.647**	-	-	-	-	-
DR subclasses	NPDR	8	**0.859**	**0.775**	0.773	**0.792**	0.782	**0.777**	-
		16	0.857	0.754	0.839	0.730	0.781	0.660	-
		32	0.763	0.763	**0.868**	0.729	**0.793**	0.647	-
	sNPDR	8	**0.741**	0.724	**0.531**	0.412	**0.464**	0.779	-
		16	**0.741**	**0.760**	0.335	**0.455**	0.385	**0.883**	-
		32	**0.741**	0.751	0.413	0.443	0.428	0.849	-
	PDR	8	**0.837**	**0.807**	0.512	0.649	**0.573**	0.906	-
		16	0.813	0.787	**0.540**	0.583	0.561	0.870	-
		32	0.834	0.804	0.446	**0.668**	0.535	**0.925**	-
Weighted metrics and Cohen's kappa for the DR subclasses		8	-	-	0.653	**0.670**	**0.658**	-	**0.575**
		16	-	-	0.650	0.631	0.636	-	0.519
		32	-	-	**0.659**	0.650	0.645	-	0.532
RVO subclasses	BRVO	8	**0.981**	0.930	0.951	**0.948**	0.950	**0.884**	-
		16	0.977	0.930	**0.978**	0.925	0.951	0.823	-
		32	0.978	**0.932**	0.970	0.934	**0.952**	0.848	-

	CRVO	8	**0.981**	0.930	**0.884**	0.890	**0.887**	0.951	-
		16	0.977	0.930	0.823	**0.944**	0.879	**0.978**	-
		32	0.978	**0.932**	0.848	0.927	0.885	0.970	-
Weighted metrics and Cohen's kappa for the RVO subclasses		8	-	-	0.930	0.930	0.930	-	**0.837**
		16	-	-	0.930	0.931	0.929	-	0.831
		32	-	-	**0.932**	**0.932**	**0.932**	-	**0.837**
AMD subclasses	Dry AMD	8	0.905	0.827	0.870	0.746	0.803	0.798	-
		16	**0.912**	0.834	**0.930**	0.733	**0.820**	0.768	-
		32	0.893	**0.841**	0.878	**0.765**	0.818	**0.815**	-
	Wet AMD	8	0.905	0.827	0.798	0.899	0.845	0.870	-
		16	**0.912**	0.834	0.768	**0.942**	0.846	**0.930**	-
		32	0.893	**0.841**	**0.815**	0.907	**0.859**	0.878	-
Weighted metrics and Cohen's kappa for the AMD subclasses		8	-	-	0.827	0.837	0.828	-	0.650
		16	-	-	0.834	**0.857**	0.835	-	0.670
		32	-	-	**0.841**	0.850	**0.842**	-	**0.678**
Mean Cohen's kappa (best epoch/100)		8	-	-	-	-	-	-	0.660 (27)
		16	-	-	-	-	-	-	**0.667** (16)
		32	-	-	-	-	-	-	0.655 (22)

Best values are highlighted in bold.
